# Synthesis and Characterization of 8-*O*-Carboxymethylpyranine (CM-Pyranine) as a Bright, Violet-Emitting, Fluid-Phase Fluorescent Marker in Cell Biology

**DOI:** 10.1371/journal.pone.0133518

**Published:** 2015-07-17

**Authors:** Eric A. Legenzov, Nathaniel D. A. Dirda, Brian M. Hagen, Joseph P. Y. Kao

**Affiliations:** Center for Biomedical Engineering and Technology, and Department of Physiology, University of Maryland School of Medicine, 111 South Penn Street, Baltimore, Maryland 21201, United States of America; Pennsylvania State Hershey College of Medicine, UNITED STATES

## Abstract

To avoid spectral interference with common fluorophores in multicolor fluorescence microscopy, a fluid-phase tracer with excitation and emission in the violet end of the visible spectrum is desirable. CM-pyranine is easily synthesized and purified. Its excitation and emission maxima at 401.5 nm and 428.5 nm, respectively, are well suited for excitation by 405-nm diode lasers now commonly available on laser-scanning microscopes. High fluorescence quantum efficiency (Q = 0.96) and strong light absorption (ε_405_ > 25,000 M^-1^cm^-1^) together make CM-pyranine the brightest violet aqueous tracer. The fluorescence spectrum of CM-pyranine is invariant above pH 4, which makes it a good fluid-phase marker in all cellular compartments. CM-pyranine is very photostable, is retained for long periods by cells, does not self-quench, and has negligible excimer emission. The sum of its properties make CM-pyranine an ideal fluorescent tracer. The use of CM-pyranine as a fluid-phase marker is demonstrated by multicolor confocal microscopy of cells that are also labeled with lipid and nuclear markers that have green and red fluorescence emission, respectively.

## Introduction

Water-soluble fluorescent markers are useful for visualizing cellular aqueous compartments and for assessing the extent to which fluid phases in different subcellular compartments mix.[1–10] The abundance of fluorophores in biological microscopy include synthetic organic molecules (e.g., fluorescein, tetramethylrhodamine, Cy5) as well as fluorescent proteins (e.g., ECFP, mCitrine, mPlum), with emissions spanning much of the visible wavelength range from ~470 nm to ~700 nm. A fluid-phase marker with emission in the violet part of the visible spectrum (~400–450 nm) would avoid spectral overlap with common fluorophores. Moreover, the wide availability of the inexpensive 405-nm diode laser as an excitation light source on laser-scanning confocal microscope makes a violet fluid-phase marker particularly useful.

We have studied the effectiveness of fusogenic peptides in promoting translocation of molecules from endo-lysosomal compartments into the cytosol following endocytosis.[11] In one recent study we used a fluorescent protein complementation assay [12], where a nonfluorescent truncated GFP lacking a C-terminal β-strand is constitutively expressed in the cytosol of CV1 cells [13–15], wherein coexpressed mCherry served as an index of protein expression. The truncated protein is nonfluorescent until the missing β-strand oligopeptide binds to it to generate a functional, fluorescent GFP. The complementary oligopeptide was co-encapsulated in liposomes with an influenza-derived fusogenic peptide [16, 17], and the liposomes entered the cell through endocytosis. To avoid spectral overlap with GFP and mCherry, we sought a violet-emitting marker that is highly water-soluble, well retained by cells, and readily excited at 405 nm.

**Fig 1:**
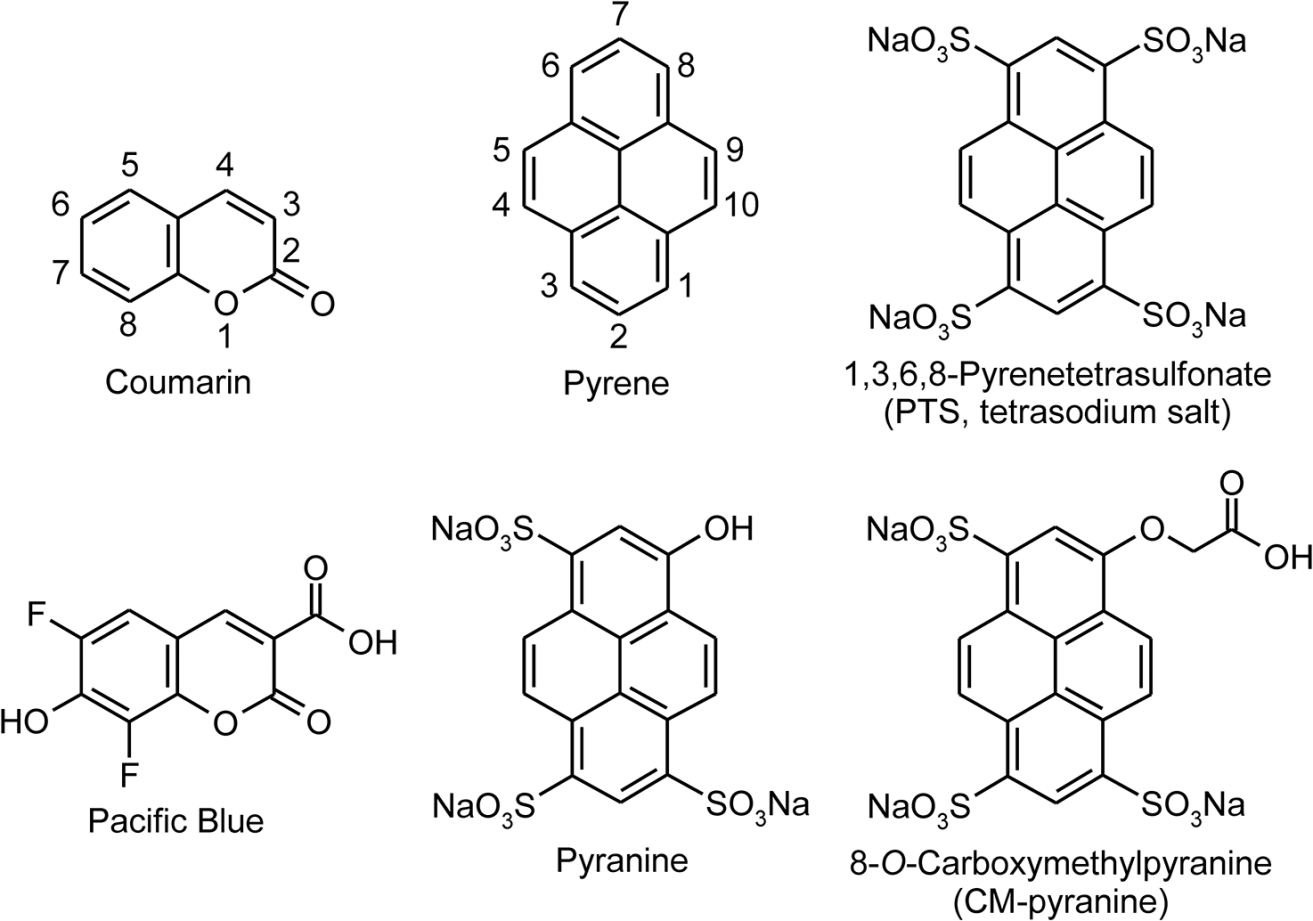
Coumarin and pyrene and water-soluble fluorophores derived from them.

Aqueous solubility is almost invariably achieved by attaching ionizable functional groups to render organic fluorophores ionically charged near physiological pH. Intracellular retention of molecular probes is enhanced by the presence of multiple ionic charges on the same molecule [18]. A survey of the literature shows that fluorophores whose excitation and emission fall in the violet range of the spectrum are typically based on coumarin or pyrene (Fig 1), whose emission maxima occur at ~380 and ~390 nm, respectively. A bathochromic shift of the excitation and emission maxima into the violet-indigo part of the visible spectrum requires one or more substituents to be added to the aromatic rings. The need for such auxochromic groups on the small coumarin ring means that it would be quite difficult to add multiple ionizable groups to the fluorophore (for example, see the already heavily-substituted structure of Pacific Blue in Fig 1). Among the pyrene-derived fluorophores, 1,3,6,8-pyrenetetrasulfonate (PTS; Fig 1), bearing four negative charges, is expected to have long intracellular retention. Unfortunately, the excitation and emission maxima of PTS, at 374 and 403 nm respectively, are too blue-shifted to be useful for excitation by 405-nm diode lasers. A pyrene-based fluorophore whose excitation and emission lie in the violet-to-blue range is pyranine (trisodium 8-hydroxypyrene-1,3,6-trisulfonate, or HPTS; Fig 1). Moreover, the presence of three negative sulfonate groups confers high aqueous solubility and should increase intracellular retention time. Pyranine, however, is a pH indicator—its excitation and emission spectra change markedly depending on the protonation state of the 8-OH group [19]. This is not ideal for a fluid-phase marker, which could be located in cellular compartments that differ widely in pH [20, 21], from ~7.2 (e.g., cytosol) to ~4.5 (e.g., lysosomes). A pyranine-derived fluorophore that is expected to be well-excited at 405 nm, and to have high aqueous solubility and good intracellular retention is 8-*O*-carboxymethylpyranine (CM-pyranine; Fig 1). In CM-pyranine, the 8-OH group, with a p*K*_a_ of 7.3, is replaced by the carboxymethoxy group, which in this molecular environment is expected to have a *pK*_a_ ≤ 3. This would ensure, at any physiologically relevant pH, 1) that CM-pyranine has four negative charges, and 2) that the excitation and emission characteristics of CM-pyranine will remain constant.

CM-pyranine itself has never been commercially available, although its use as a synthetic intermediate to proprietary fluorescent labeling reagents of the Cascade Blue family has been disclosed [22, 23]. In the present study, we describe a modified synthesis and purification of CM-pyranine, characterize its fluorescence properties and photostability, assess its retention inside cells, and use multi-color laser-scanning confocal microscopy to demonstrate its use as an aqueous marker to track fluid phase movement between cellular compartments.

## Materials and Methods

### General

For organic synthesis, reagents and solvents of ACS or higher grade from commercial suppliers were used without further purification. UV-visible absorption spectroscopy was performed on a Cary 300 spectrophotometer (Varian, Inc., Palo Alto, CA); a background buffer comprising 100 mM KCl and 10 mM HEPES (2-[4-(2-hydroxyethyl)piperazin-1-yl]ethanesulfonic acid), at pH 7.2, was used. NMR spectra were recorded at 400 MHz (400 MR, Varian Instruments, Palo Alto, CA) in D_2_O and were referenced to the residual solvent peak (δ_H_ 4.79); coupling constants are in Hz. Abbreviations used to indicate the multiplicity of NMR resonances: s = singlet; d = doublet; t = triplet; m = multiplet. High-resolution mass spectrometry (electrospray ionization) was carried out at the mass spectrometry facility in the Chemistry and Biochemistry Department at The University of Maryland (College Park, MD).

For cell biology experiments, CV1 cells were from ATCC (Manassas, VA), media were from GIBCO (Life Technologies, Grand Island, NY), biochemical reagents were from Sigma-Aldrich (St. Louis, MO), and lipids were from Avanti Polar Lipids (Alabaster, AL). Monomeric INF7 peptide (H_2_N-GLFEAIEGFIENGWEGMIDGWYGC-CO_2_H) was synthesized by GenScript USA, Inc. (Piscataway, NJ). The monomer was converted to the disulfide-linked dimer essentially as previously described [11], by dissolving the peptide in water along with 7 molar equivalents of K_2_CO_3_ and maintaining the solution at 45–50°C and in the presence of pure oxygen for 4 hr. The disulfide-linked dimer of INF7 is used in all biological studies presented here. For simplicity, hereafter the term “INF7” refers to the dimer.

### Chemistry

Trisodium 8-(methoxycarbonylmethoxy)pyrene-1,3,6-trisulfonate (**1**)

A solution of trisodium 8-hydroxypyrene-1,3,6-trisulfonate (0.510 g, 0.98 mmol; Sigma-Aldrich, St. Louis, MO) in 20 mL methanol was heated to reflux, followed by the addition of methyl bromoacetate (0.564 g, 0.342 mL, 3.6 mmol) and *N*,*N*-diisopropylethylamine (0.349 g, 0.470 mL, 2.7 mmol) in 3 aliquots over 5 h. Reflux was maintained for 3 additional h; thereafter the reaction mixture was cooled to room temperature and filtered. The filtrate was reduced to dryness by rotary evaporation; the residue was stirred in 9.5 mL isopropanol for 30 min and collected by filtration. The recovered yellow powder was dried under vacuum. The crude product was purified on a Sephadex LH-20 column with water as eluant. Three bands were distinguishable: a minor, fast-eluting band, a middle band, and a slower-eluting band. The fast, minor band was discarded, while the latter two bands were collected separately. Lyophilization of the middle band yielded a yellow powder (0.390 g, 0.65 mmol, 67%). ^1^H-NMR (D_2_O; full spectrum provided as S1 Fig): 9.17 (s, 1H), 9.13 (d, 1H, *J* = 9.8 Hz), 9.06 (d, 1H, *J* = 9.8 Hz), 8.99 (d, 1H, *J* = 10.2 Hz), 8.85 (d, 1H, *J* = 9.4 Hz), 8.23 (s, 1H), 5.25 (s, 2H), 3.86 (s, 3H); HRMS (ESI) *m/z*: [M + H – 3Na]^2-^ calcd for C_19_H_12_O_12_S_3_, 263.9745; found 263.9754. Lyophilization of the slow band gave an amber-yellow powder (0.10 g) whose ^1^H-NMR and mass spectra were essentially identical to those of the middle band. The pure yellow material from the middle band was used for the next reaction.

Trisodium 8-(carboxymethoxy)pyrene-1,3,6-trisulfonate, or CM-pyranine (**2**)

Compound **1** (0.359 g, 0.60 mmol) was dissolved in 2.4 M HCl (60 mL) and maintained at 90°C for 24 h. The solution was reduced to near-dryness by rotary evaporation. The residue was diluted with water and again reduced by rotary evaporation; this process was repeated twice. The resulting gum was dissolved in water; the solution was frozen and lyophilized to yield a light yellow powder (0.338 g, 96%). ^1^H-NMR (D_2_O; full spectrum provided as S1 Fig): 9.16 (s, 1H,) 9.12 (d, 1H, *J* = 9.8 Hz), 9.10 (d, 1H, *J* = 9.8 Hz), 9.05 (d, 1H, *J* = 9.8 Hz), 9.03 (d, 1H, *J* = 9.4 Hz) 8.25 (s, 1H), 5.21 (s, 2H); HRMS (ESI) *m/z*: [M + H – 3Na]^2-^ calcd for C_18_H_10_O_12_S_3_, 256.9667; found 256.9673).

### Fluorescence Spectroscopy

All fluorescence spectra were acquired on a SPEX spectrofluorimeter (Model CM1T10I, HORIBA Scientific, Edison, NJ). For pH studies, CM-pyranine was used at low concentration in cuvettes with 1-cm path length, and fluorescence emission was acquired in the right-angle detection mode. CM-pyranine (5 × 10^−7^ M) was dissolved in a solution containing 150 mM KCl, and a mixed-buffer system comprising 5 mM each of acetate, phosphate, and borate (all as the appropriate sodium salt). The pH of the solution was decremented by addition of 10% HCl, and excitation and emission spectra were recorded at each pH. After background subtraction, each spectrum was corrected for the minor volume change resulting from HCl addition. The pH range examined spanned 3.85 to 8.2.

Solutions of the Na salt of CM-pyranine, ranging in concentration from 50 μM to 20 mM, were used in experiments aimed at detecting excimer emission. The samples were measured using a microcuvette with a path length of 50 μm (NSG Precision Cells, Farmingdale, NY), and fluorescence emission was acquired in the front-face mode. After background subtraction, each emission spectrum was smoothed with a 7-point quadratic Savitzky-Golay algorithm [24] and then normalized to the maximum intensity. The normalized spectra were overlaid for graphical presentation. To better visualize excimer emission, difference spectra were generated as follows. Normalized spectra of samples at 0.25, 0.5, and 1.0 mM, which showed no excimer emission and good signal-to-noise, were averaged. This averaged spectrum was subtracted from the normalized spectra at 2.5, 5.0, 10, and 20 mM to generate the difference spectra at those concentrations.

### Determination of the Quantum Efficiency of Fluorescence Emission

The fluorescence quantum efficiency of CM-pyranine was determined relative to that of the standard, quinine; standard methodology was used [25, 26]. Quinine (≥ 98.0%, Sigma-Aldrich, St. Louis, MO) was dissolved in 1.0 *N* H_2_SO_4_ to a concentration of 50 mM. CM-pyranine was dissolved in deionized water to a concentration of 50 mM; the 50 mM solution was diluted 10-fold to yield a 5 mM working stock solution. The background buffers used for spectrofluorimetry were: for quinine, 1.0 *N* H_2_SO_4_; for CM-pyranine, 100 mM KCl buffered with 10 mM HEPES (pH 7.4). The appropriate fluorophore stock solution was added to each background buffer to yield a known, low absorbance at 372 nm (measured *A*_372_ = 0.101 for both quinine and CM-pyranine); each sample was then diluted 5-fold for spectroscopy. Spectra were recorded with excitation at 372 nm, and emission was scanned over the range 377–700 nm; all spectra were acquired at 25°C. Identical scans were performed on the background buffers. All spectra were smoothed with a 7-point quadratic Savitsky-Golay algorithm. After subtraction of the background spectra, the sample spectra were integrated; the ratio of the integrals was 1.750 (CM-pyranine/quinine). The quantum efficiency of quinine is *Q* = 0.546 [25, 27]; applying the ratio of the integrals gives *Q* = 0.96 for CM-pyranine.

In relative determinations of quantum efficiency against a reference standard, significant differences in the refractive indices of the standard and sample solutions must be taken into account. In the present case, however, the refractive indices of the two solutions (1 *N* H_2_SO_4_ and 100 mM KCl) are expected to differ by ~3 parts per thousand, which can be considered negligible [28–30]. It is also important to note that the reported *Q* for quinine was at infinite dilution, and the Stern-Volmer constant for quinine self-quenching is 14.5 M^-1^ [27]. At the quinine concentration used in the present measurements (~8 μM), self-quenching would be expected to reduce the quantum efficiency by ~1 part per 10,000—a negligible effect. Finally, the present measurements were made in air-equilibrated aqueous solution, containing ~200 μM dissolve O_2_, which has negligible effect on quinine emission [27]. The determined *Q* for CM-pyranine therefore applies to aqueous solutions equilibrated with air.

### Assessing Self-quenching of CM-pyranine and Fluorescein by 2-Photon Microscopy

Measurements of self-quenching were performed on an inverted laser-scanning confocal microscope (LSM 510 NLO, Carl Zeiss GmbH, Jena, Germany) equipped with an infrared-transmitting oil-immersion objective (EC Plan-Neofluar, 40×, N.A. 1.3), and a mode-locked titanium-sapphire (Ti:S) laser (Chameleon Ultra II, Coherent, Inc., Santa Clara, CA) for 2-photon excitation; fluorescence emission passed through a 658-nm short-pass filter before photometric quantitation. Droplets (3 μL) of solutions of the Na salt of CM-pyranine or fluorescein (ranging from 50 μM to 20 mM, adjusted to pH > 10 by addition of Na_2_CO_3_ to ensure that all fluorophores are fully ionized) were deposited on No. 1 glass coverslips mounted in custom Petri dishes; sufficient USP-grade mineral oil was added to keep the droplets submersed to prevent evaporation. Ti:S output at 800 nm was used to excite both fluorophores. The focal point was positioned within a droplet, at ~4–5 μm above the upper surface of the coverslip. This ensured that the focal point was inside the “bulk” solution, but minimized the thickness of solution that fluorescence photons must traverse, thus essentially eliminating any inner-filter effect that could reduce fluorescence through reabsorption at high fluorophore concentrations. For acquiring the background signal, the focal point was positioned outside aqueous droplets, at ~4–5 μm above the coverslip. Every image (or frame) was line-averaged 4 times during acquisition. For each data point, 4 successive frames of 512 × 512 pixels (225 × 225 μm) were acquired and averaged; duplicate measurements were performed at each concentration.

Intensity was digitized at 16-bit depth to give a grey-level range of 0–65,535. For each series of fluorophore solutions, the detector gain was adjusted so that the solution showing the highest fluorescence intensity yielded images with essentially no saturated pixels. A region-of-interest (ROI) measuring 150 × 125 μm was positioned at the center of each image; the mean intensity of all pixels within the ROI was computed. The mean background intensity determined from the background images was subtracted from all mean fluorescence intensities measured on fluorophore solutions. The background-corrected mean fluorescence intensity was plotted as a function of fluorophore concentration and analyzed by least-squares curve fitting.

The use of 2-photon microscopy for assessing self-quenching deserves some explanation. In conventional fluorescence spectroscopy, the sample thickness, and thus the optical path traversed by the excitation light beam, has macroscopic dimensions. Consequently, at high fluorophore concentrations, the optical density of the sample can be so high that the excitation beam is continuously attenuated by absorption as it traverses the sample. As a result, the excitation light intensity decreases exponentially along the beam path, and the measured fluorescence becomes a nonlinear function of fluorophore concentration. Performing the measurement on a 2-photon microscope eliminates this type of “inner filter” effect: A molecule that can absorb a single photon at wavelength λ is generally incapable of single-photon absorption at wavelength 2λ (i.e., the molar absorptivity, or extinction coefficient, at 2λ is zero). In 2-photon microscopy, excitation light at 2λ is focused through an objective lens into the sample. The current experimental parameters (NA = 1.3, refractive index = 1.33) allows estimation of the 2-photon focal volume as 0.049 μm^2^, or ~50 femtoliters [31]. In the minuscule focal volume, the light intensity is sufficiently high to enable “simultaneous” absorption of *two* photons at 2λ by the same molecule [32], which is energetically equivalent to absorption of a single photon at wavelength λ. Since 2-photon absorption occurs only within the focal volume, and outside the focal volume photons at 2λ cannot be absorbed, attenuation of the excitation light by the inner filter effect does not occur.

Whether excited through 1- or 2-photon absorption, a fluorophore generally emits fluorescence identically [33]. If the excitation and emission spectra overlap significantly, then fluorescence emitted by one fluorophore can be reabsorbed by other fluorophores. This is also an inner filter effect that depends on fluorophore concentration. By focusing the excitation beam a few μm into the fluorophore solution, fluorescence emitted from within the 2-photon focal volume traverses only the few μm of fluorophore solution before collection by the objective lens. This minimizes attenuation of emitted fluorescence by the inner filter effect.

Finally, we chose to perform 2-photon excitation at the wavelength where Ti:S output is essentially maximum—800 nm, which is expected to work well for CM-pyranine, whose 1-photon excitation maximum is ~400 nm. For fluorescein, with 1-photon excitation maximum at 489 nm, one might have expected 2-photon excitation at 800 nm to be suboptimal. Contrary to expectation, the 2-photon absorption cross-section of fluorescein shows a broad maximum around 800 nm [34]. Therefore, 2-photon excitation at 800 nm works well for both fluorescein and CM-pyranine.

### Assessing Photostability of CM-pyranine Relative to Fluorescein

Rates of photobleaching of CM-pyranine and fluorescein were compared on a laser-scanning confocal microscope (LSM 5 *Live DuoScan*, Carl Zeiss) equipped with an oil-immersion objective (EC Plan-Neofluar, 40×, NA 1.3, DIC). Solutions used for photobleaching experiments contained 100 μM fluorescein or 100 μM CM-pyranine in water, both as the fully neutralized Na salt. Minute (< 1 μL) droplets of the aqueous solution were deposited on a No. 1 glass coverslip mounted in a custom-made chamber by tapping a 10-μL pipet tip containing fluorophore solution on the glass surface. The droplets were immediately submersed under USP-grade mineral oil to prevent evaporation. With the thin tip of a gel-loading pipette, the droplets were scrambled under the mineral oil to generate microscopic droplets of varying sizes. The micro-droplets adhere to the glass coverslip and form circular discs. Discs approximating mammalian cell dimensions (18–67 μm in diameter) were chosen for photobleaching experiments. At these diameters, the disc thickness is expected to be < 50 μm, which means that the optical density was < 0.04, ensuring negligible attenuation of the light beam.

Discs of CM-pyranine and fluorescein solution were repeatedly scanned at 405 nm and 489 nm, respectively, at a rate of one frame per 0.514 sec. In both cases, the pinhole was set at 1 Airy unit, and the power was set to be nominally 0.5 mW of laser output. On the LSM 5 *Live* microscope, the 405-nm and 489-nm laser outputs are each attenuated by a different acousto-optic tunable filter before launch into separate fiberoptic conduits for delivery to the scanner. Therefore, nominally identical laser output power does not guarantee identical power delivery at the objective lens. Measured with a laser power meter (LabMaster Ultima, Coherent Inc., Santa Clara, CA) coupled with a silicon sensor (LM-2 VIS, Coherent Inc.) at the front face of the objective, the actual powers at 405 and 489 nm were 12.4 and 55.8 μW, respectively. Finally, the extinction coefficients of the fluorophores at the respective photobleaching wavelengths are also important for comparison of photobleaching rates. The relevant values are ε _405_ = 25,780 M^-1^cm^-1^ for CM-pyranine, and ε _489_ = 76,660 M^-1^cm^-1^ for fluorescein.

For analysis, a circular region-of-interest (ROI) measuring approximately a tenth of the total area of the imaged disc was placed at the center of the disc. The raw fluorescence signal in each frame was measured as the average intensity of all pixels within the ROI. The background in each frame, measured as the average pixel intensity in a second ROI placed outside the imaged disc, was subtracted from the raw signal in each frame to give the background-corrected fluorescence intensity. A plot of intensity vs. time was used to determine the half-life of the fluorophore in the photobleaching experiment.

### Cell Culture

All cell culture media products were from GIBCO (Life Technologies, Grand Island, NY). The CV-1 cell line [13], purchased from American Type Culture Collection (Manassas, VA), were cultured in polystyrene flasks at 37°C in Dulbecco’s Modified Eagle Medium (DMEM), supplemented with 10% v/v fetal bovine serum, 2 mM glutamine, 100 U/mL penicillin, and 100 μg/mL streptomycin, and equilibrated with humidified air containing 5% CO_2_. For microscopy studies, cells were detached from the culture flasks by brief treatment with trypsin-EDTA (0.25%) solution, suspended in supplemented DMEM and plated onto 25-mm round No. 1 glass coverslips; the plated cells were maintained in culture and used for experiments over the next 3 days.

### Half-life of Intracellular Retention of CM-pyranine at Physiologic Temperature

CM-pyranine or carboxyfluorescein (CF) were introduced into cells by the scratch-loading technique [35]. A coverslip bearing CV1 cells were mounted in a custom-made flow chamber. To maintain cell health during scratch loading required a high-K^+^, low-Na^+^, low-Ca^2+^ saline containing (in mM) 145 KCl, 8 MgCl_2_, 10 KH_2_PO_4_, 1 K_2_H_2_EGTA, 1 NaATP, and 1 NaGTP, adjusted to pH 7.4 with 1 M KOH. Immediately before scratch loading, a 250-μL of high-K^+^ saline containing 2 mM CM-pyranine and 2 mM CF (both as Na salt) was place onto the cells. One tip of a pair of microdissection forceps was drawn rapidly across the cell-bearing surface of the coverslip; cells closely bordering the resulting scratch took up fluorophores from the saline. Thereafter the fluorophore-containing saline was removed by suction. The cells were immediately rinsed 10 times with 250-μL aliquots of FluoroBrite DMEM (Gibco) containing 10 mM HEPES (FluoroBrite-HEPES) and then immersed in FluoroBrite-HEPES for confocal microscopy. The flow chamber was positioned on the microscope, and continuously superfused with FluoroBrite-HEPES at ~0.5 mL/min at 37 C.

CM-pyranine and CF were scanned at 405 nm and 489 nm, respectively, with the pinhole set for 10.6-μm optical sections. Images were acquired at 1-minute intervals; the entire sequences comprised 164 images. Light exposure of the sample was reduced 300-fold relative to that in the photobleaching experiment. The experiment was terminated when the intracellular carboxyfluorescein fluorescence decayed to less than 10% of initial value. Background fluorescence was measured in an area of the coverslip devoid of cells. The background-subtracted fluorescence from 17 contiguous cells enclosed by a single ROI were averaged. Plots of CM-pyranine and CF fluorescence intensity vs. time were used to estimate the intracellular half-lives of the fluorophores.

A control experiment demonstrating that both 405-nm light and CM-pyranine are benign is shown as S2 Fig. Comparative images of CV1 cells simultaneously loaded with three different fluorophores are provided as S3 Fig.

### Liposome Preparation

Liposomes comprised 1,2-distearoylphosphatidylcholine (DSPC), cholesterol (Chol), L-α-phosphatidylserine (PS; porcine brain isolate), and 1,2-dioleoyl-*sn*-glycero-3-phosphatidylethanolamine-*N*-(carboxyfluorescein) ammonium salt (CF-PE) in the molar ratio 3:2:0.3:0.003 (DSPC:Chol:PS:CF-PE). A solution of 10 μmol phospholipid in 100 μL ethanol was injected into 1 mL of rapidly stirred aqueous solution to be encapsulated (see below). The mixture was extruded 11 times through a 100-nm porosity filter membrane (Nucleopore Track-etch, Whatman, a division of GE Healthcare, Piscataway, NJ) in a Mini Extruder (Avanti Polar Lipids) to yield a suspension of liposomes. All solutions and the extruder were maintained at >55°C to ensure fluidity of the lipid phase. Encapsulated solution contained (in mM): 10 CM-pyranine, 73.3 NaCl, 2 KCl, 0.25 MgCl_2_, and 0.34 CaCl_2_. Liposomes were purified by gel filtration on Sephadex G-50 resin (~4 g; GE Healthcare) packed into a FlexColumn (15 × 300 mm; Kimble-Kontes, Vineland, NJ), and equilibrated with Dulbecco’s phosphate-buffered saline (DPBS). The band of liposomes on the column could be tracked visually by the blue fluorescence of the encapsulated CM-pyranine. Gel filtration yielded ~4 mL of purified liposome suspension in DPBS (final phospholipid concentration ~2 μmol/mL), which was stored at 4°C until use.

### Cellular Uptake of Fluorophores through Fluid-phase Endocytosis

CV-1 cells on glass coverslips were incubated for 90 min under 5% CO_2_ in air at 37°C with 8 mM CM-pyranine, 5 mM propidium iodide, and 93 μM dimeric INF7 peptide in a 4:1 (v/v) mixture of FluoroBrite and the purified liposome suspension in DPBS (described above). The negatively charged headgroups of PS stimulate CV1 cells to endocytose the liposomes avidly [14, 15], with concomitant endocytosis of the extracellular fluid. Thereafter, the incubation medium was removed, and the cells were rinsed 10 times with FluoroBrite DMEM containing 20 mg/mL bovine serum albumin, and once with plain FluoroBrite DMEM. The cells were then transferred into plain FluoroBrite-HEPES for confocal microscopy.

To activate the INF7 peptide, CV1 cells mounted on the confocal microscope were transiently exposed to 1% acetic acid in FluoroBrite for 160 sec and then returned to FluoroBrite-HEPES. Acetic acid, being neutral and membrane-permeant, rapidly enters cells and acidifies intracellular compartments [11]. The cells were imaged immediately before and after the transient acidification.

### Scanning Confocal Fluorescence Microscopy

Cellular imaging experiments were performed on a laser scanning confocal microscope (model LSM 5 *Live DuoScan*, Carl Zeiss Microscopy, GmbH, Jena, Germany) fitted with an oil-immersion objective (EC Plan-Neofluar, 40×, NA 1.3, DIC). The following excitation and emission wavelengths were used: for CM-pyranine, excitation at 405 nm, emission 415–505 nm; for CF-PE, excitation at 488 nm, emission 495–555 nm; for propidium iodide, excitation at 561 nm, emission 580 nm (longpass).

### Data Analysis and Presentation

ImageJ software [36] was used for image analysis; cropping and thresholding of images for presentation were performed with Photoshop (Adobe Systems, San Jose, CA). Numerical data reduction and analysis were performed with Origin software (OriginLab, Northampton, MA). Numerical results are given as mean ± std. dev.

## Results and Discussion

### Chemistry

**Fig 2:**
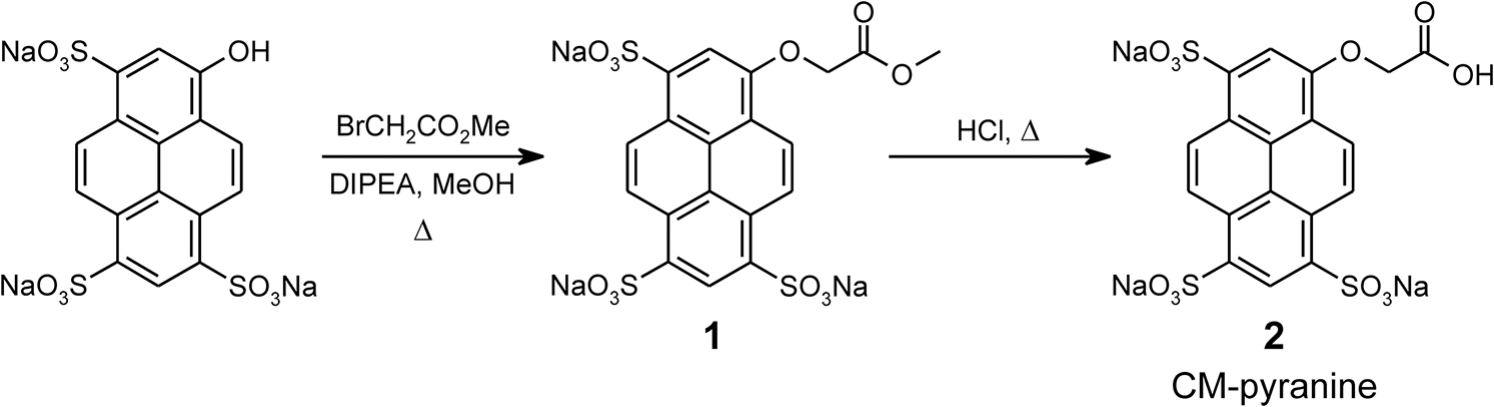
Synthesis of CM-pyranine.

The synthesis of CM-pyranine is outlined in Fig 2. Commercially available pyranine was alkylated with methyl bromoacetate in refluxing methanolic solution essentially as described [23]. Chromatography of the crude product on Sephadex LH-20 with water as eluant resolved 8-*O*-methoxycarbonylmethylpyranine (**1**) as the major product (see Experimental section for details of chromatographic separation). Methyl ester **1** was quantitatively hydrolyzed with 2.4 M aqueous HCl at 90°C; evaporation of the reaction mixture followed by lyophilization afforded pure CM-pyranine (**2**). The advantage of the acid hydrolysis of **1** is that all components of the reaction other than the desired product **2** are volatile, and are easily removed by evaporation and lyophilization.

**Fig 3:**
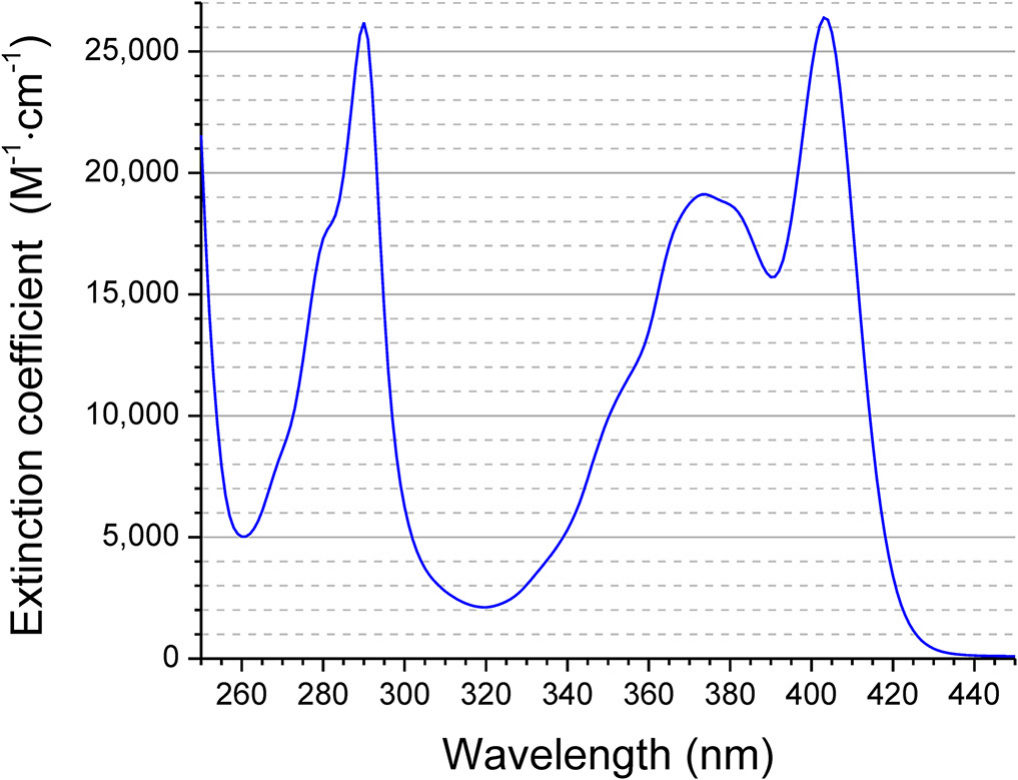
UV-visible absorption spectrum of CM-pyranine. Spectrum acquired in buffer comprising 100 mM KCl, 10 mM HEPES, pH 7.2.

The UV-visible absorption spectrum of CM-pyranine is shown in Fig 3. The absorption maximum is at 403 nm, with an extinction coefficient of ε = 2.64 × 10^4^ M^-1^cm^-1^. The quantum efficiency of fluorescence emission of CM-pyranine was determined relative to that of quinine, and has the value *Q* = 0.96 in air-saturated aqueous solution.

### Fluorescence properties of CM-pyranine

#### pH independence of CM-pyranine fluorescence

**Fig 4:**
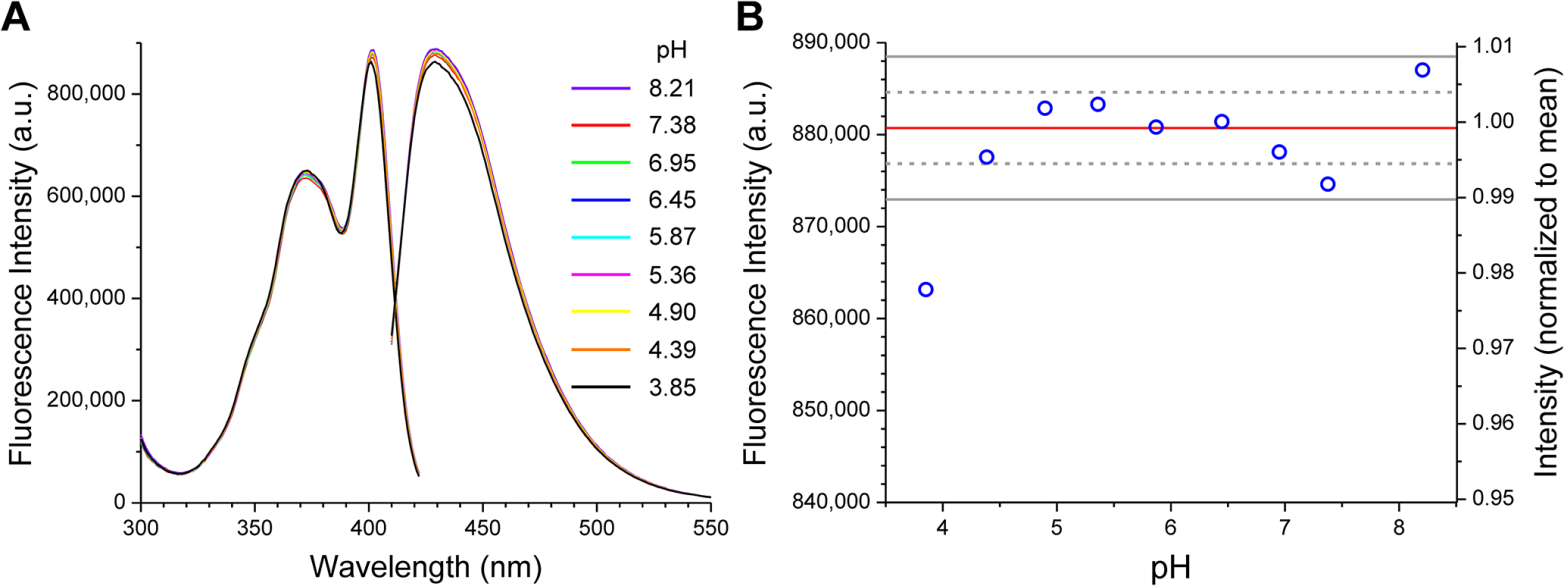
Effect of pH on CM-pyranine fluorescence. **(A)** Excitation and emission spectra of CM-pyranine at various pH values. The excitation maximum is at 401.5 nm, and the emission maximum is at 428.5 nm. **(B)** Maximum fluorescence intensity recorded for each sample plotted as function of pH. Red line marks the mean of the 8 points spanning the pH range 4.39–8.21; gray dashed and solid lines mark ± 1 SD and ± 2 SD from the mean, respectively. The intensity at pH 3.85 differs from the mean by more than 4 SDs, although in relative terms, it is only ~2% less than the mean (refer to normalized scale on the right axis).

As discussed above, the carboxyl group in CM-pyranine is expected to have *pK*_a_ ≤ 3. This implies that the spectroscopic properties of CM-pyranine should be completely pH-independent at pH > 4. This expectation is confirmed by fluorescence spectra of CM-pyranine recorded over the pH range 3.85 to 8.2 (Fig 4A). The excitation and emission spectra are essentially unchanged over this wide pH range (Fig 4A). The excitation maximum occurs at 401.5 nm, and the emission maximum is at 428.5 nm. The excitation and emission intensities recorded at pH 3.85 are statistically lower than those recorded at pH > 4 (Fig 4B), but in relative terms, the difference amounts to only ~2% (Fig 4B, reading from right axis). Thus, the fluorescence of CM-pyranine is invariant at pH > 4. Since pH in cellular compartments can range from a low of ~4.5 in lysosomes, to ~7.2 in the nucleo-cytoplasm, to a high of ~8 in the mitochondrial matrix [21], the pH-independence of CM-pyranine means that it is a good fluid-phase marker irrespective of the cellular compartment.

#### CM-pyranine exhibits minimal excimer emission

**Fig 5:**
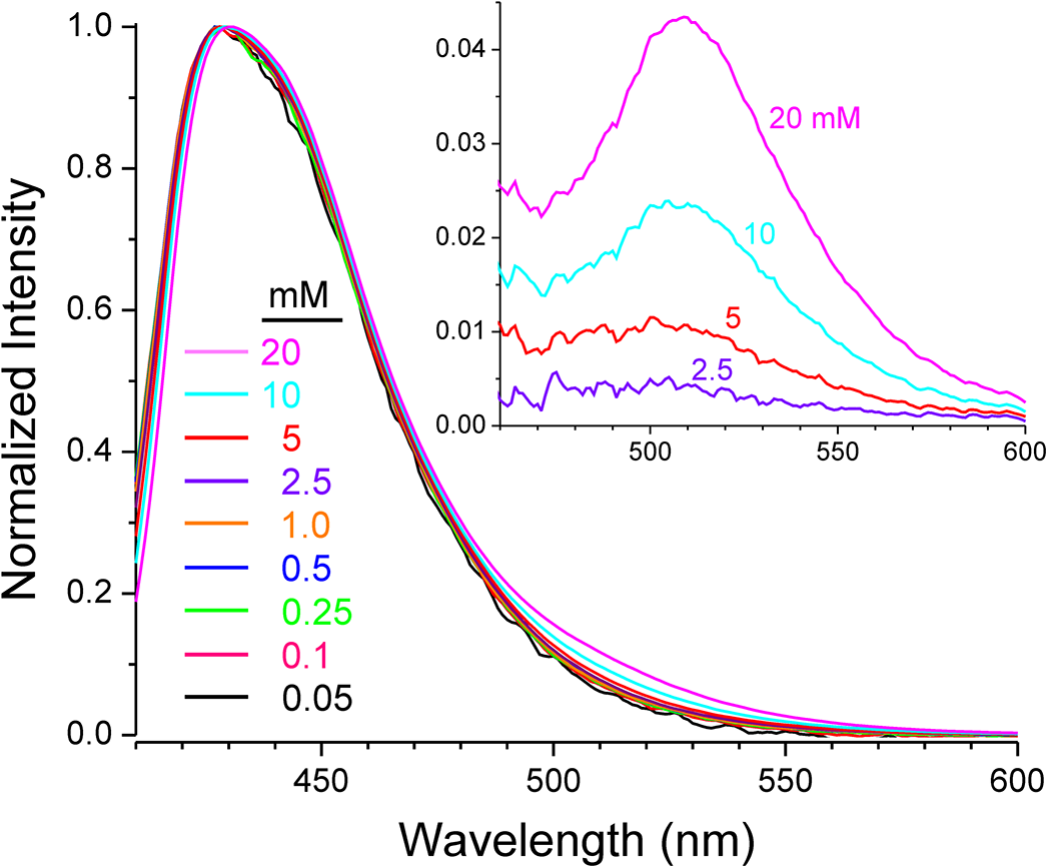
CM-pyranine fluorescence emission as function of concentration. CM-pyranine concentration for each spectrum is indicated by the color of each curve. Difference spectra (inset) show the gradual appearance of minor excimer emission at CM-pyranine concentrations > 1 mM.

CM-pyranine is a pyrene bearing multiple substituents. Pyrene itself exhibits the well-known phenomenon of excimer emission: an excited pyrene molecule can interact with a second molecule of pyrene to form a transient excited-state dimer (excimer); emission from the excimer regenerates two ground-state molecules. Pyrene excimer emission is strongly red-shifted (by ~100 nm) relative to monomer emission [37]. Importantly, excimer emission, owing to the need for dimer formation, is concentration-dependent. With increasing concentration, excimer emission grows at the expense of monomer fluorescence emission. Such behavior is undesirable for a fluid-phase fluorescent marker. To ascertain the propensity of CM-pyranine for excimer emission, emission spectra were recorded over a wide concentration range. Superposition of the normalized spectra (Fig 5) shows that with the 400-fold increase in concentration from 50 μM to 20 mM, the emission spectra remain essentially similar. Closer inspection suggests that in the region around 500 nm, a slight increase in emission may be apparent at concentrations above 1 mM. Difference spectra (Fig 5 insert) confirm the appearance of emission centered at ~508 nm. Importantly, however, even at 20 mM (a concentration unlikely to be used in biology), the increased emission at 508 nm is only ~4% relative to the monomer fluorescence. At the somewhat more biologically realistic concentration of 5 mM (e.g., when encapsulated in liposomes), the increase is only ~1%. This is in marked contrast to the behavior of the parent pyrene fluorophore, whose excimer emission dwarfs monomer emission at mM concentrations [37]. Therefore, in practical terms, the propensity of CM-pyranine for excimer emission is negligible. The key difference between CM-pyranine and pyrene is that CM-pyranine bears four negative charges and is thus well-solvated by water molecules, whereas pyrene is a very hydrophobic, uncharged molecule. The electrostatic repulsion between highly charged CM-pyranine molecules, and the fact that good solvation by water minimizes the tendency to self-aggregate, together make excimer formation energetically unfavorable.

#### CM-pyranine does not self-quench at millimolar concentrations

In concentration-dependent self-quenching, the quantum efficiency of fluorescence emission diminishes at high concentrations of fluorophore. Although different microscopic mechanisms have been proposed to underlie self-quenching of various fluorophores [38–40], the phenomenon of reduced quantum efficiency at high concentration is consistently observed. In order to assess self-quenching of CM-pyranine, it was necessary to quantify fluorescence emission of CM-pyranine over a wide concentration range (5 ×10^−5^–2 ×10^−2^ M). For comparison, fluorescence emission of fluorescein over the same concentration range was also examined. All the experiments on self-quenching were performed on a 2-photon laser-scanning microscope, because 2-photon microscopy has key advantages over conventional fluorescence spectroscopy for studying emission from concentrated fluorophore solutions (see Experimental Section for detailed explanation).

**Fig 6:**
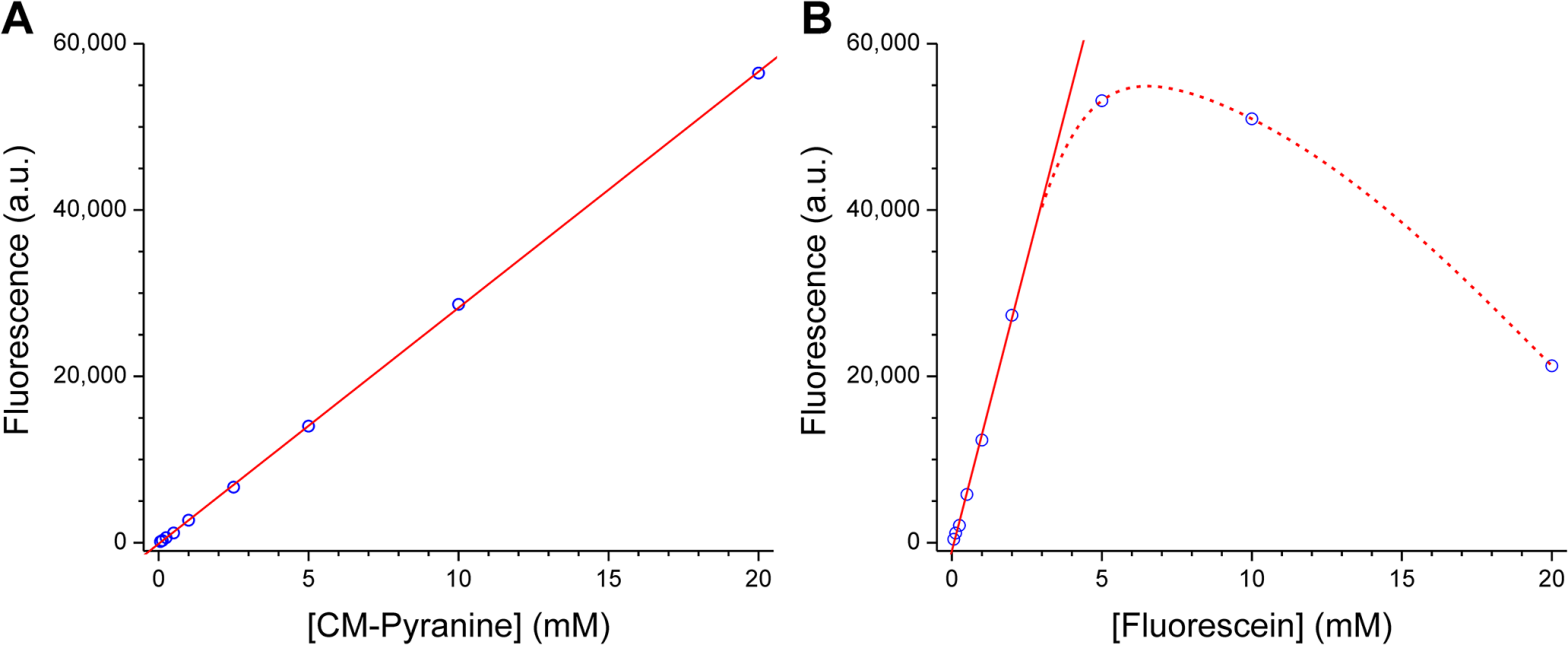
Concentration dependence of 2-photon-excited fluorescence emission for CM-pyranine and fluorescein. 2-photon-excited fluorescence from solutions of each fluorophore at various concentrations were imaged on a laser-scanning microscope. After background subtraction, the average fluorescence intensity in each image was plotted against concentration (see Experimental Section for details). Each data point is the average of 4–8 replicate measurements; all error bars are smaller than the symbol size. **(A)** Fluorescence vs. concentration for CM-pyranine. The red line is the linear least-squares fit of the data. Linearity is observed over the entire concentration range examined (up to 20 mM). **(B)** Fluorescence vs. concentration for fluorescein. The solid red line is the linear least-squares fit of the data up to 2 mM. The dashed red line is a visual aid to show nonlinear behavior at high concentrations of fluorescein.

Two-photon excitation of CM-pyranine was performed at the wavelength where the titanium:sapphire laser has essentially maximal output—800 nm. This corresponds to a single-photon wavelength of 400 nm, which closely matches the single-photon excitation maximum of CM-pyranine. Fig 6A shows the concentration dependence of CM-pyranine fluorescence. The dependence is linear over the entire concentration range examined (0.05–20 mM). In contrast, the concentration dependence for fluorescein is obviously nonlinear over the same concentration range (Fig 6B), a consequence of its propensity for self-quenching [27, 41]. Since self-quenching could have dynamic and static components, two differences between CM-pyranine and fluorescein may be relevant for the observed difference. First, under our experimental conditions, CM-pyranine is a tetra-anion and fluorescein is a di-anion; therefore, electrostatic repulsion makes encounter or association between CM-pyranine molecules less likely than between fluorescein molecules. Second, the Stokes shift for CM-pyranine is somewhat larger than that for fluorescein (27 nm vs. 23 nm); therefore there is less overlap between the excitation and emission spectra of CM-pyranine, which is less conducive to self-quenching.

### Photostability of CM-pyranine

**Fig 7:**
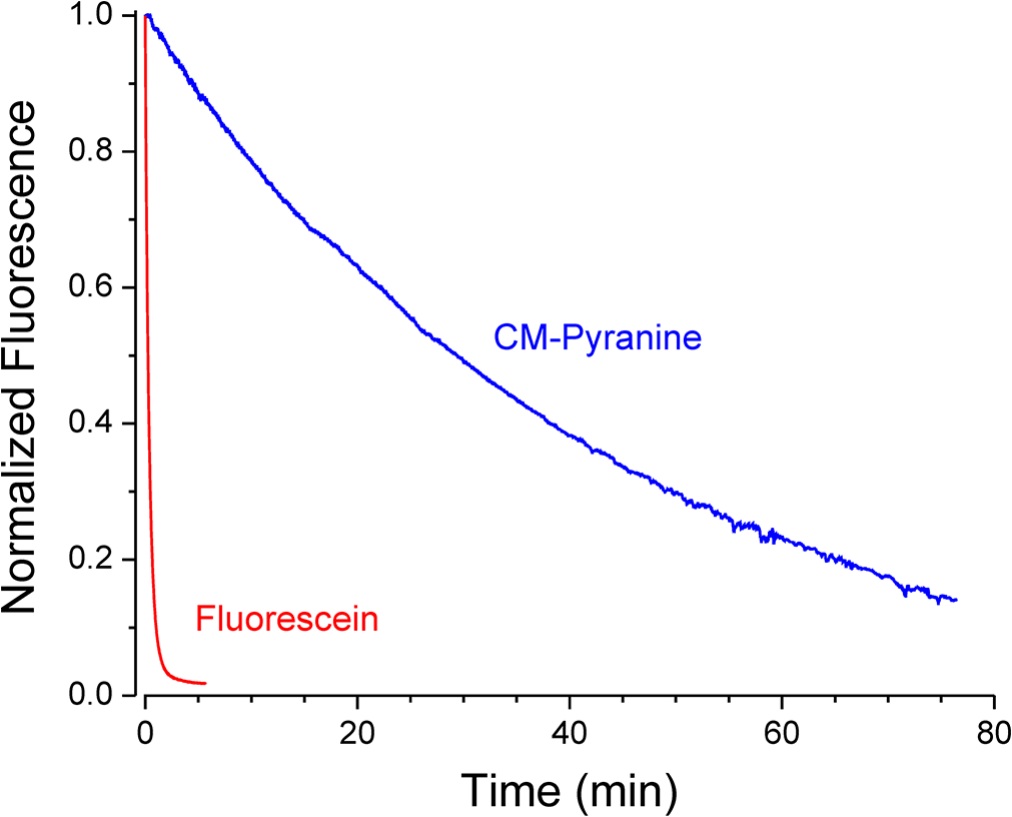
CM-pyranine is resistant to photobleaching. Microdroplets of CM-pyranine and fluorescein were photobleached at 405 and 489 nm, respectively (see Experimental section for details). Progress of photobleaching was monitored by confocal imaging of the microdroplets. The fluorescence at any time is normalized to the intensity at *t* = 0. Two representative time courses are shown. For all measurements performed, mean half-lives were 1510 ± 230 sec (or 25.1 ± 3.8 min; n = 3) and 11.2 ± 3.1 sec (n = 8) for CM-pyranine and fluorescein, respectively.

The ideal fluorescent tracer is photostable; i.e., the fluorophore is not readily destroyed by photobleaching. Microdroplets of 100 μM CM-pyranine solution comparable in size to mammalian cells were photobleached at 405 nm using a laser-scanning confocal microscope; the progress of photobleaching was conveniently monitored by confocal imaging of the droplets. For comparison, parallel experiments were performed on microdroplets of 100 μM fluorescein solution at 489 nm. Typical time courses for photobleaching of CM-pyranine and fluorescein are shown in Fig 7. Under the photobleaching conditions of the experiment, the half-life of CM-pyranine was 1510 ± 230 sec (or 25.1 ± 3.8 min; n = 3), whereas the half-life of fluorescein was 11.2 ± 3.0 sec (n = 8). The relative susceptibility of the two fluorophores to photobleaching is not simply given by the ratio of the two observed half-lives, because a fair comparison requires that the same amount of energy be absorbed by the two samples. With the two fluorophores at the same concentration, the energy absorbed by each sample depends on the power (*P*) incident on the sample and the extinction coefficient (ε) of the fluorophore at the wavelength of the incident light. The normalization factor is thus (*P*ε)_fluorescein_/(*P*ε)_CM-pyranine_ = 13.4 (*P* and ε values specified in Experimental Section). Applying the normalization factor to the ratio of the half-lives shows that fluorescein photobleaches 10 times more rapidly than CM-pyranine. This represents the relative susceptibility to photobleaching on an energy-absorbed basis. An alternative way to view the results is based on the number of photons absorbed: Equal amounts of *energy* at 408 and 489 nm represent different number of *photons*. Since each photon carries energy *E* = *hc*/λ (where *h* is Planck’s constant, *c* is the velocity of light, and λ is the wavelength), the proportionality factor is just the ratio of wavelengths: 489/405 = 1.207. Applying this factor gives the relative bleaching rate of fluorescein to CM-pyranine as 8.4-fold faster on a per-photon basis. CM-pyranine is thus much more photostable than fluorescein, one of the most commonly used fluorescent markers in cell biology.

### Intracellular retention of CM-pyranine

**Fig 8:**
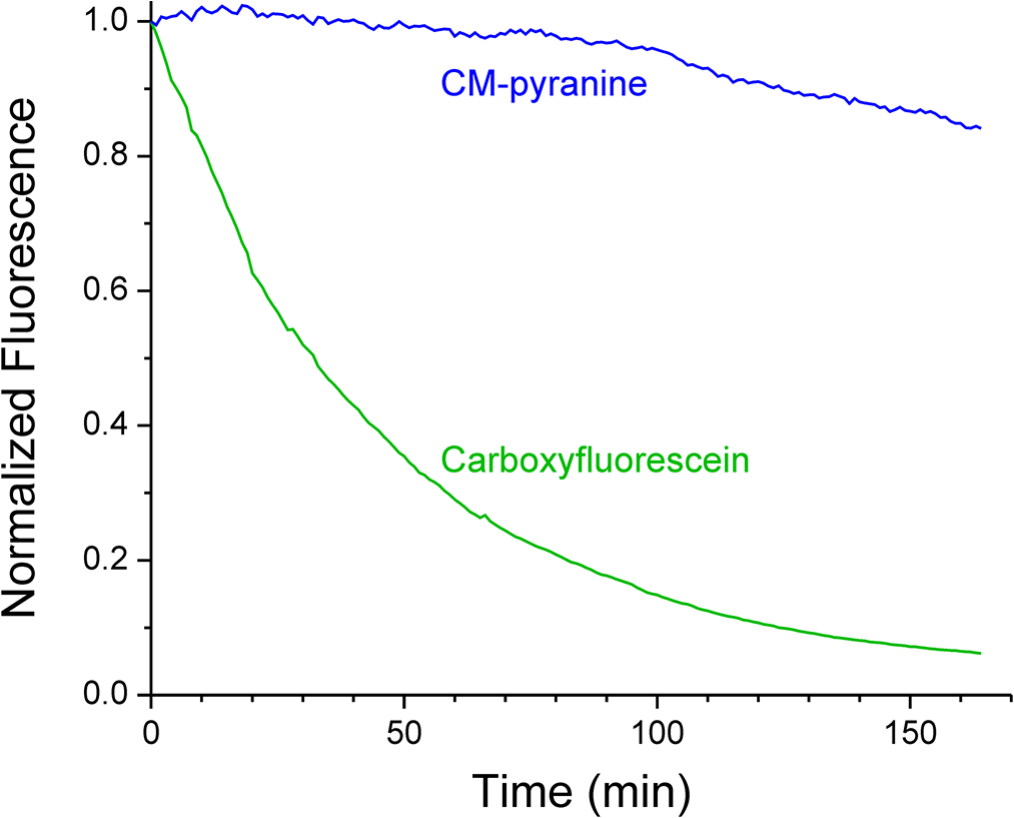
CM-pyranine is well-retained in cells. CV1 cells were loaded with CM-pyranine and carboxyfluorescein (CF) by the scratch loading technique. The cells were continuously superfused with medium at 37°C. **(A)** The retention of the fluorophores inside cells was monitored by confocal imaging at 1 min intervals. The fluorescence intensity at any time is normalized to the intensity at *t* = 0. The time courses shown are the average of 17 contiguous cells enclosed within a single region-of-interest. Red dots mark the times (0 and 124 min) at which the images shown in panel B were taken. **(B)** Images of CF (green) and CM-pyranine (blue) in one cluster of cells acquired at 0 and 124 min; the times correspond to the points marked by red dots on the graph in panel A.

Negatively charged xenobiotic organic molecules are extruded from cells by organic anion transporters [42–44]. The extrusion mechanisms are steeply temperature-dependent and become very efficient at physiologic temperature [18, 45, 46]. Among other determinants, the number of ionic charges on a molecule strongly influences the extrusion rate [18]. To compare kinetics of cellular extrusion of CM-pyranine, which bears 4 negative charges, and carboxyfluorescein (CF), which bears 3 negative charges at physiologic pH, the two fluorophores were introduced into CV1 cells by the scratch-loading technique [35]. The cells were then perfused with medium at 37°C. The loss of fluorescent marker over time was monitored by confocal imaging at long intervals (1 min); light exposure of the sample was reduced 300-fold relative to that in the photobleaching experiment. The average time courses of marker loss from 17 cells are shown in Fig 8. These results show that at the time when 90% of CF was lost (~124 min), only 10% of the CM-pyranine had been lost. CM-pyranine is thus well-retained intracellularly even at physiologic temperature and is, therefore, an excellent fluid-phase fluorescent marker for long-term cellular imaging experiments.

### Use of CM-pyranine as fluid-phase marker in multicolor imaging microscopy

To demonstrate the utility of CM-pyranine as a fluid-phase marker in multi-color imaging, CV1 cells were incubated at 37°C in FluoroBrite containing CM-pyranine, propidium iodide (PI), as well as nanoliposomes incorporating phosphatidylserine (PS), which confers negative surface charge, and carboxyfluorescein-tagged phosphatidylethanolamine (CF-PE), which acts as a fluorescent tracer for the lipid phase. The incubation medium also contained 93 μM INF7, an influenza-derived fusogenic peptide that, at acidic pH, promotes translocation of fluid-phase molecules from the endosomal system into the nucleocytoplasm [16, 17]. CV1 cells avidly take up liposomes bearing negative lipid head groups [14, 15] through receptor-mediated endocytosis. As a consequence, robust uptake of the PS-containing nanoliposomes also promotes fluid-phase endocytosis, enabling CM-pyranine, PI, and INF7 to enter the endosomal system. After a 90-min incubation, the cells were transferred into FluoroBrite-HEPES and mounted on the confocal microscope. To activate INF7 peptide by acidification, the cells were transiently exposed to 1% acetic acid in FluoroBrite, and then returned to FluoroBrite-HEPES. The three fluorophores, CM-pyranine, CF-PE, and PI in the CV1 cells were imaged immediately before and after the transient acidification.

**Fig 9:**
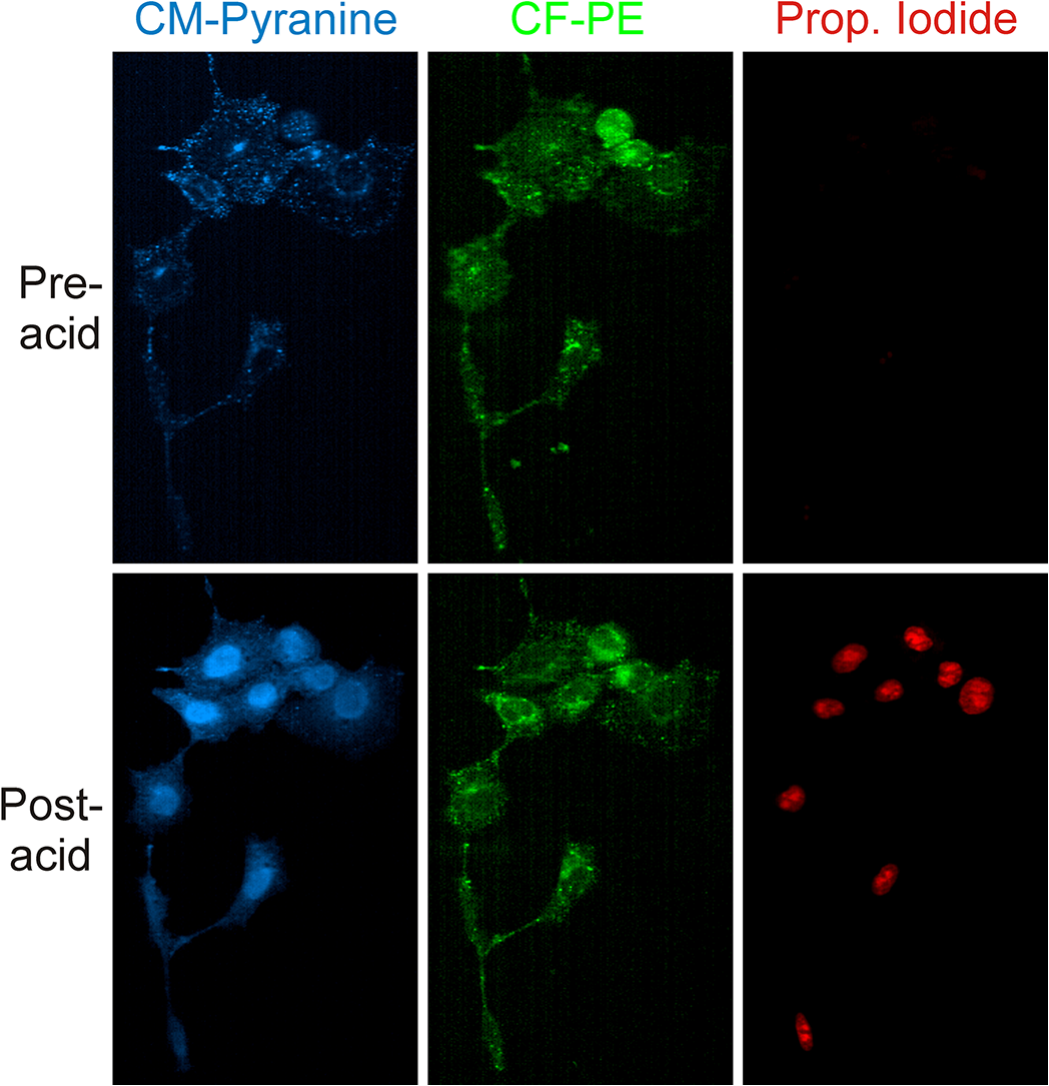
INF7-promoted translocation of fluid phase fluorophores from the endosomal system into the nucleocytoplasm. CV1 cells were allowed to endocytose CF-PE-tagged liposomes bearing negative surface charge, which promoted concomitant fluid-phase endocytosis of CM-pyranine, propidium iodide (PI), and pH-sensitive fusogenic INF7 peptide in the extracellular medium. Transient exposure to 1% acetic acid activated the INF7 peptide to enable fluid-phase molecules to move from the endosomal system into the nucleocytoplasm. Top and bottom rows show, respectively, images of the cells acquired before and after activation by acid.

Fig 9 shows the images acquired before and after acidification. Before acidification, CM-pyranine (blue) is confined to punctate structures, consistent with its localization in the fluid phase of the endosomal system. CF-PE (green), being a marker of the lipid phase of the endocytosed liposomes, is also associated with the endosomal system. PI (red), being membrane-impermeant, is likewise confined to the fluid phase of the endosomal system following endocytosis. Before acidification, endosomally-associated PI shows very weak fluorescence, because PI becomes brightly fluorescent only upon intercalation into nucleic acids. Upon acidification, activation of INF7 promotes movement of molecules from the fluid-phase of the endosomal system into the nucleocytoplasm; this has two visible consequences. First, as CM-pyranine permeates the nucleocytoplasm, its fluorescence becomes more spatially uniform throughout the cells. Second, PI entering the nucleocytoplasm can intercalate into DNA in the nucleus, and the consequent increase in red fluorescence highlights the nuclei in the cells. Finally, CF-PE, being associated with the membrane lipid phase, remains largely unaffected by the action of INF7.

## Conclusions

CM-pyranine is easy to synthesize and purify. Its fluorescence quantum efficiency is very high, and its fluorescence characteristics are pH-independent above pH 4, making CM-pyranine useful as a fluid-phase marker in all cellular compartments. CM-pyranine shows no self-quenching and negligible excimer emission even at many-millimolar concentrations. CM-pyranine is very resistant to photobleaching and is retained intracellularly for long periods of time even at physiological temperature. With excitation and emission maxima at the extreme blue end of the visible spectrum, CM-pyranine can be used in combination with diverse synthetic and genetically-encodable fluorophores with minimal spectral overlap. Furthermore, CM-pyranine is well excited by 405-nm diode lasers now commonly available on laser-scanning microscopes. These characteristics make CM-pyranine an ideal fluid-phase fluorescent tracer in cell biology.

## Supporting Information

S1 Fig NMR spectra of CM-pyranine methyl ester and free acid. (PDF)

S2 Fig CM-pyranine and 405-nm light are benign. (PDF)

S3 Fig Images of cells loaded with 3 water-soluble fluorophores. (PDF)
