# Supplementary material for: Synthesis and Characterization of 8-O-Carboxymethylpyranine (CM-Pyranine) as a Bright, Violet-Emitting, Fluid-Phase Fluorescent Marker in Cell Biology
Source: PLoS One. 2015 Jul 17;10(7):e0133518. doi: 10.1371/journal.pone.0133518 (PMC4505926; doi:10.1371/journal.pone.0133518)
Supplement: S1 Fig — (PDF) [file pone.0133518.s001.pdf]

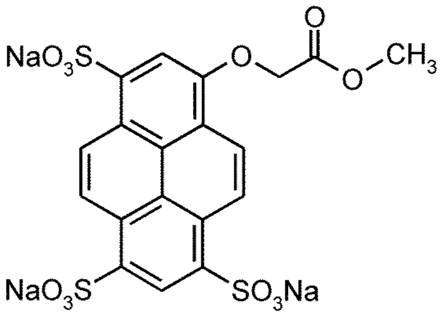

in D_2O
referenced to residual
solvent resonance

INDEX	FREQUENCY	PPM	HEIGHT
1	3605.2	9.019	28.5
2	3590.3	8.981	15.7
3	3580.1	8.956	22.2
4	3562.9	8.913	16.6
5	3553.5	8.889	22.6
6	3534.4	8.841	23.4
7	3524.6	8.817	16.7
8	3485.1	8.718	16.0
9	3475.7	8.695	13.2
10	3233.5	8.089	32.4
11	2045.3	5.116	44.1
12	1853.2	4.636	1260.8
13	1483.8	3.712	108.8

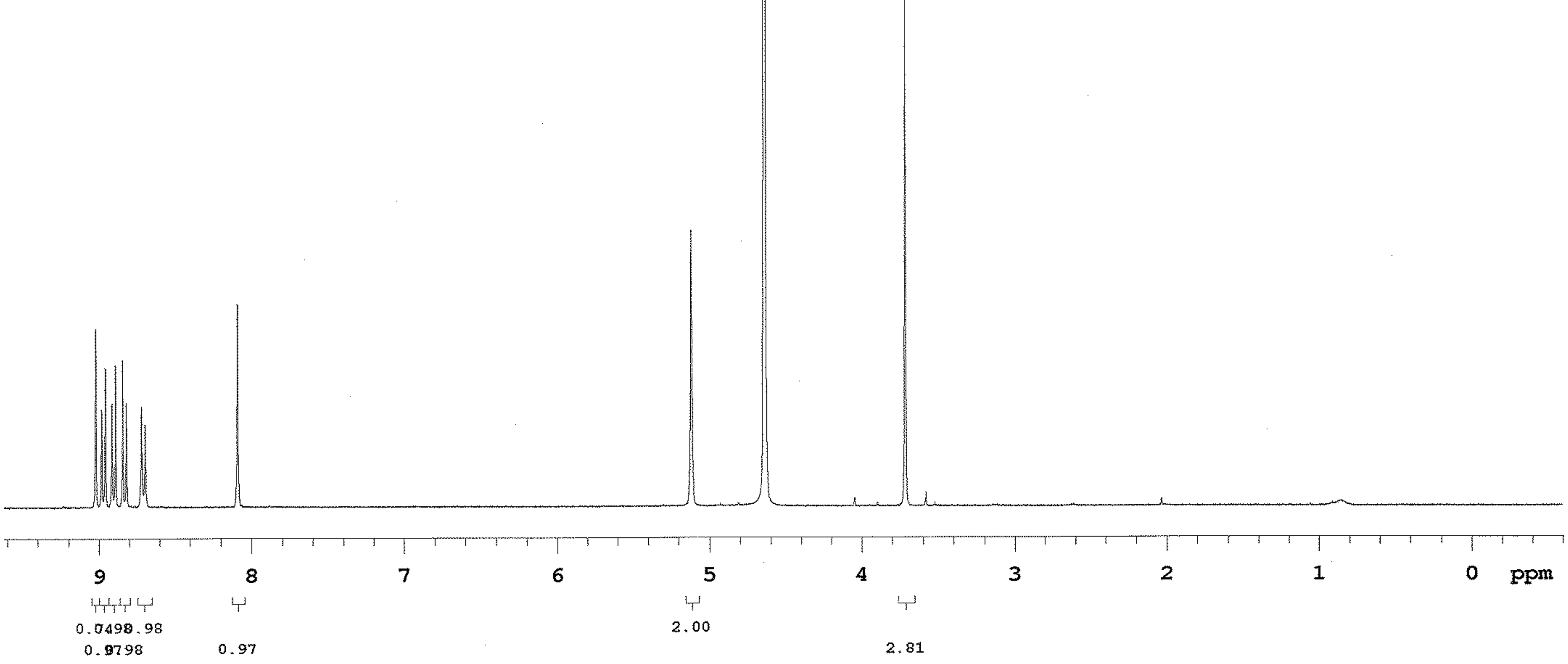

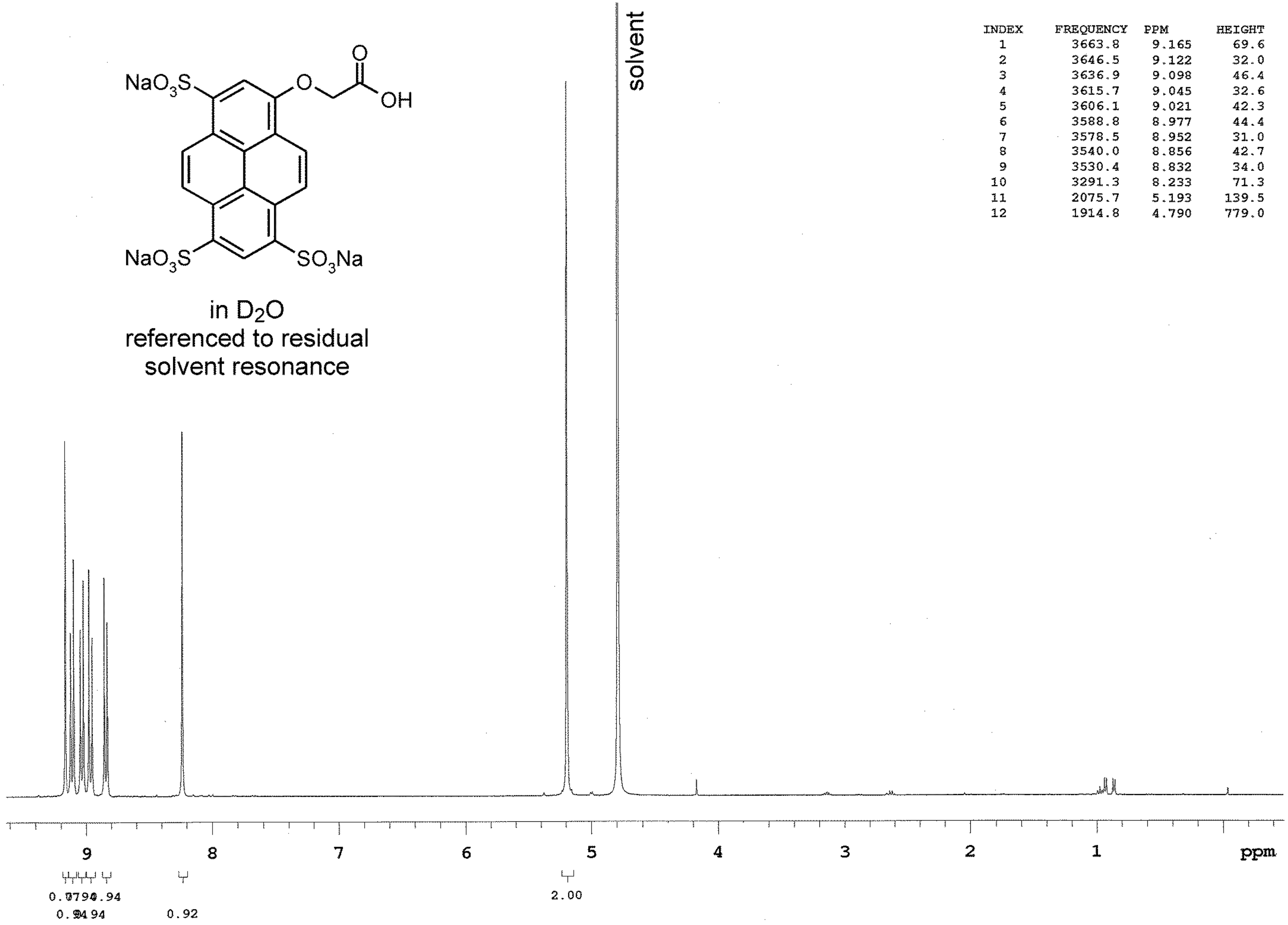

in D_2O
referenced to residual
solvent resonance

INDEX	FREQUENCY	PPM	HEIGHT
1	3663.8	9.165	69.6
2	3646.5	9.122	32.0
3	3636.9	9.098	46.4
4	3615.7	9.045	32.6
5	3606.1	9.021	42.3
6	3588.8	8.977	44.4
7	3578.5	8.952	31.0
8	3540.0	8.856	42.7
9	3530.4	8.832	34.0
10	3291.3	8.233	71.3
11	2075.7	5.193	139.5
12	1914.8	4.790	779.0
