# Supplementary material for: Synthesis and Characterization of 8-O-Carboxymethylpyranine (CM-Pyranine) as a Bright, Violet-Emitting, Fluid-Phase Fluorescent Marker in Cell Biology
Source: PLoS One. 2015 Jul 17;10(7):e0133518. doi: 10.1371/journal.pone.0133518 (PMC4505926; doi:10.1371/journal.pone.0133518)

CM-pyranine and 405-nm light are benign and well tolerated by cells

CV1 cells were scratch-loaded with CM-pyranine and carboxyfluorescein (CF) and imaged exactly as described in the Methods section, under the subheading **"Half-life of Intracellular Retention of CM-pyranine at Physiologic Temperature"**. A series of 448 images were acquired. The first and last images of the series are shown in the figure below (Frame 0 and Frame 448, respectively). No morphological signs of cytotoxicity are apparent at the end of the sequence. We conclude that 405-nm light per se and fluorescence excitation of CM-pyranine under normal imaging conditions are both benign and well tolerated by cells.

Note that as demonstrated in the photobleaching studies, CF fluorescence was much dimmer in the last frame compared to the first, while there was no comparable reduction in CM-pyranine fluorescence. This is consistent with the results of the photobleaching studies—that CM-pyranine is more resistant to photobleaching.

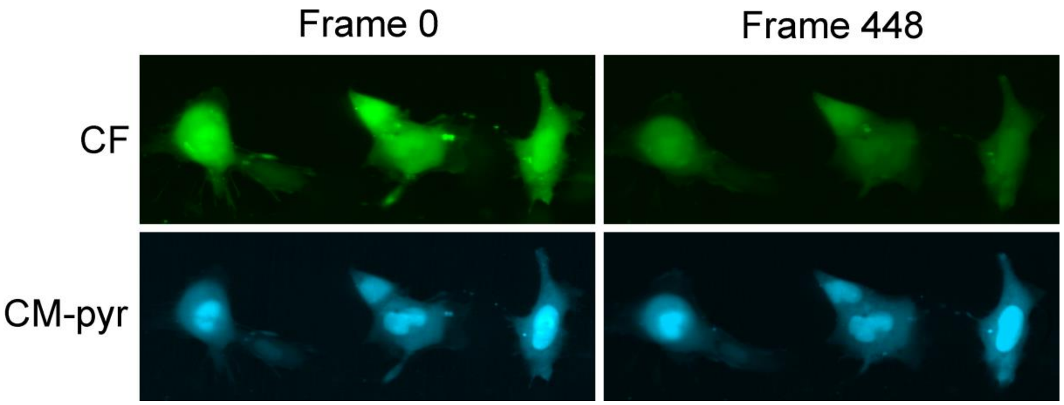

Supplement: S2 Fig — (PDF) [file pone.0133518.s002.pdf]
