# Supplementary material for: Synthesis and Characterization of 8-O-Carboxymethylpyranine (CM-Pyranine) as a Bright, Violet-Emitting, Fluid-Phase Fluorescent Marker in Cell Biology
Source: PLoS One. 2015 Jul 17;10(7):e0133518. doi: 10.1371/journal.pone.0133518 (PMC4505926; doi:10.1371/journal.pone.0133518)
Supplement: S3 Fig — (PDF) [file pone.0133518.s003.pdf]

Comparative images of cells loaded with three different water-soluble fluorophores

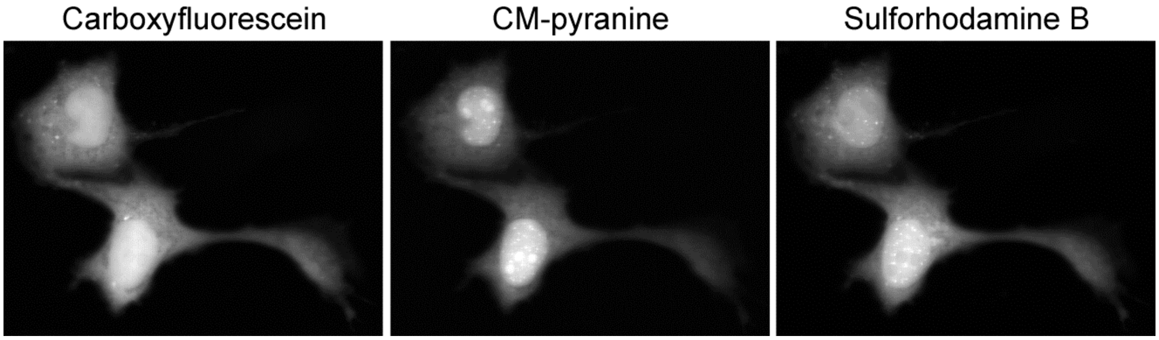

All three fluorophores distribute throughout the cells, with higher fluorescence in the nucleus, as is typically observed with low-molecular-weight synthetic fluorophores. The structures of the three fluorophores are shown below.

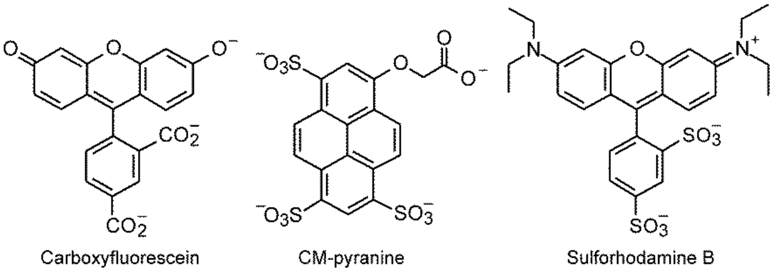

CM-pyranine, carboxyfluorescein (CF), and sulforhodamine B (SR-B) were introduced into cells by the scratch-loading technique.[1] A coverslip bearing CV1 cells were mounted in a custom-made flow chamber. To maintain cell health during scratch loading required a high-K^+, low-Na^+, low-Ca^{2+} saline containing (in mM) 145 KCl, 8 $MgCl_2$, 10 KH_2PO_4, 1 K_2H_2EGTA, 1 NaATP, and 1 NaGTP, adjusted to pH 7.4 with 1 M KOH. Immediately before scratch loading, a 250-µL of high-K^+ saline containing 2 mM each of CM-pyranine, CF, and SR-B was place onto the cells. One tip of a pair of microdissection forceps was drawn rapidly across the cell-bearing surface of the coverslip; cells closely bordering the resulting scratch took up fluorophores from the saline. Thereafter the fluorophore-containing saline was removed by suction. The cells were immediately rinsed 10 times with 250-µL aliquots of FluoroBrite DMEM (Gibco) containing 10 mM HEPES (FluoroBrite-HEPES) and then immersed in FluoroBrite-HEPES for confocal microscopy. The flow chamber was positioned on the microscope, and continuously superfused with FluoroBrite-HEPES at ~0.5 mL/min at 24 °C. CM-pyranine, CF, and SR-B were excited at 405 nm, 489 nm, and 561 nm respectively, with the pinhole set for 10.6-µm optical sections.

[1] McNeil, P. L. (2001) Direct introduction of molecules into cells, *Curr Protoc Cell Biol Unit 20.1*.
